# Genistein: A Potential Natural Lead Molecule for New Drug Design and Development for Treating Memory Impairment

**DOI:** 10.3390/molecules27010265

**Published:** 2022-01-01

**Authors:** Shivkanya Fuloria, Muhamad Azrul Amir Yusri, Mahendran Sekar, Siew Hua Gan, Nur Najihah Izzati Mat Rani, Pei Teng Lum, Subban Ravi, Vetriselvan Subramaniyan, Abul Kalam Azad, Srikanth Jeyabalan, Yuan Seng Wu, Dhanalekshmi Unnikrishnan Meenakshi, Kathiresan V. Sathasivam, Neeraj Kumar Fuloria

**Affiliations:** 1 Faculty of Pharmacy, AIMST University, Bedong 08100, Malaysia; shivkanya_fuloria@aimst.edu.my (S.F.); aphdukm@gmail.com (A.K.A.); 2 Department of Pharmaceutical Chemistry, Faculty of Pharmacy and Health Sciences, Royal College of Medicine Perak, Universiti Kuala Lumpur, Ipoh 30450, Malaysia; mazrul.yusri@s.unikl.edu.my (M.A.A.Y.); mahendransekar@unikl.edu.my (M.S.); peiteng1013@gmail.com (P.T.L.); 3 School of Pharmacy, Monash University Malaysia, Bandar Sunway 47500, Malaysia; gan.siewhua@monash.edu; 4 Faculty of Pharmacy and Health Sciences, Royal College of Medicine Perak, Universiti Kuala Lumpur, Ipoh 30450, Malaysia; najihah.izzti@gmail.com; 5 Department of Chemistry, Karpagam Academy of Higher Education, Coimbatore 641021, India; ravisubban@rediffmail.com; 6 Faculty of Medicine, Bioscience and Nursing, MAHSA University, Jalan SP 2, Bandar Saujana Putra, Jenjarom Selangor, Shah Alam 42610, Malaysia; drvetriselvan@mahsa.edu.my; 7 Department of Pharmacology, Sri Ramachandra Faculty of Pharmacy, Sri Ramachandra Institute of Higher Education and Research (DU), Chennai 600116, India; srikanth.j@sriramachandra.edu.in; 8 Centre for Virus and Vaccine Research, School of Medical and Life Sciences, Sunway University, Subang Jaya 47500, Malaysia; sengwu_21@yahoo.com; 9 Department of Biological Sciences, School of Medical and Life Sciences, Sunway University, Subang Jaya 47500, Malaysia; 10 College of Pharmacy, National University of Science and Technology, Muscat 130, Oman; dhanalekshmi@nu.edu.om; 11 Faculty of Applied Sciences, AIMST University, Bedong 08100, Malaysia

**Keywords:** genistein, isoflavone, memory impairment, neuroprotection, phytomedicine

## Abstract

Genistein is a naturally occurring polyphenolic molecule in the isoflavones group which is well known for its neuroprotection. In this review, we summarize the efficacy of genistein in attenuating the effects of memory impairment (MI) in animals. Scopus, PubMed, and Web of Science databases were used to find the relevant articles and discuss the effects of genistein in the brain, including its pharmacokinetics, bioavailability, behavioral effects, and some of the potential mechanisms of action on memory in several animal models. The results of the preclinical studies highly suggested that genistein is highly effective in enhancing the cognitive performance of the MI animal models, specifically in the memory domain, including spatial, recognition, retention, and reference memories, through its ability to reduce oxidative stress and attenuate neuroinflammation. This review also highlighted challenges and opportunities to improve the drug delivery of genistein for treating MI. Along with that, the possible structural modifications and derivatives of genistein to improve its physicochemical and drug-likeness properties are also discussed. The outcomes of the review proved that genistein can enhance the cognitive performance and ameliorate MI in different preclinical studies, thus indicating its potential as a natural lead for the design and development of a novel neuroprotective drug.

## 1. Introduction

**Figure 1:**
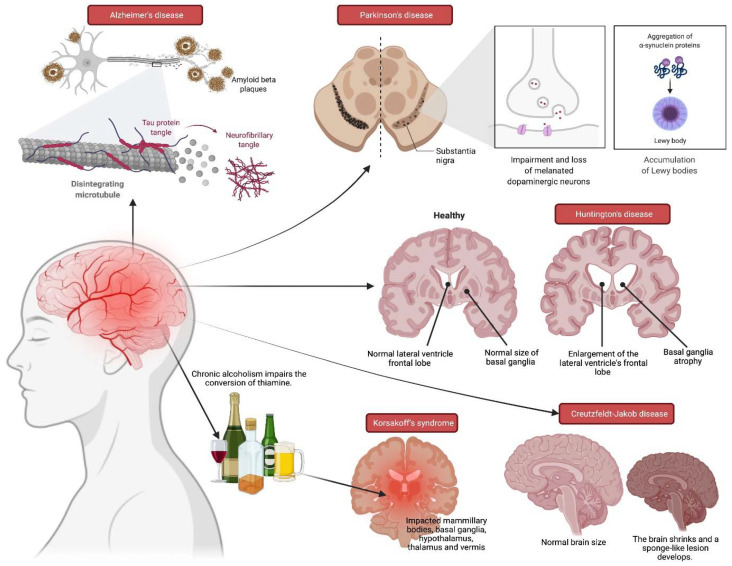
Disorders of the brain that can lead to memory impairment.

Memory is a state of gaining, retaining, and retrieving information which includes all knowledge gained throughout one’s experience such as truths that are known, incidents remembered, and abilities nurtured throughout one’s life. Two main types of memory are declarative and non-declarative memories, with the former being the daily memories while the latter mainly consist of memories retrieved reflexively [1]. Memory disorders, often known as memory impairment (MI), are key indicators for diagnosing certain aetiologies associated to syndromes. Some cases in point include Alzheimer’s, Parkinson’s, Huntington’s, Korsakoff’s, and Creutzfeldt-Jakob diseases (Figure 1) [2,3,4,5]. MI mainly affects the declarative memory, as is the case with amnesia and dementia, but this is not always the case with the latter, since dementia is defined as the decline in two or more areas of cognition. In other words, dementia is not limited to declarative memory disorder alone since it also affects other parts of memory [1].

Dementia can affect memory in both primary and secondary ways. Primary memory disability may include the decline in declarative memory in which Alzheimer’s disease is a good demonstration since declarative memory is one of the areas of cognitive that suffer from the decline. On the other hand, the secondary manner in which memory ability is affected is when there are cognitive deficits that can impede memory performance, e.g., attentional disorder dementia that may hinder multiple aspects of memory performance [1].

Currently, there is no confirmed treatment that can totally attenuate the development of MI. Nonetheless, memory improvement therapies are important for maintaining a patient’s cognitive function with the aim of combating MI risk factors. Estrogen, which is a reproductive hormone, has a broad spectrum of action with its neuroprotective role. Nevertheless, its potential as a neuroprotective agent can be ameliorated by the proliferation and oncogenic effects on certain cells, causing the need for the development of selective estrogen receptor modulators (SERMs), including the naturally occurring phytoestrogens [6] like genistein.

**Figure 2:**
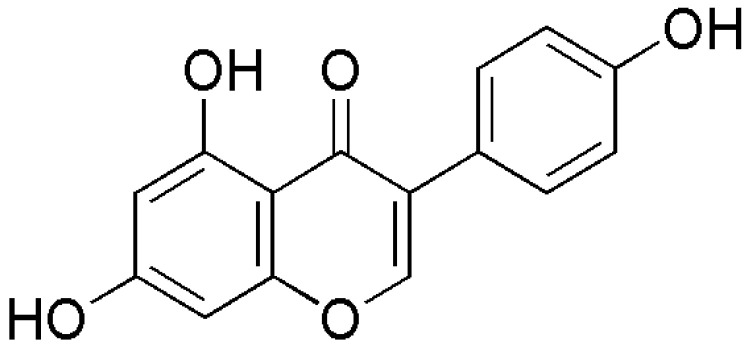
Chemical structure of genistein.

Genistein is an isoflavone (Figure 2) predominantly found in *Glycine max* (soybean) extract among many other sources, such as legumes, peanuts, and green peas. Genistein is produced following metabolism of the biologically active glycoside genistin [7]. Since many traditional Asian foods are made from soybean, e.g., natto, tofu, and sufu [8], Asian countries recorded a relatively high amount of genistein (25–30 mg/day) intake when compared with Western countries (2 mg/day). In fact, fermentation of soybeans is also one of the best ways to release genistein other than digestion [9].

**Figure 3:**
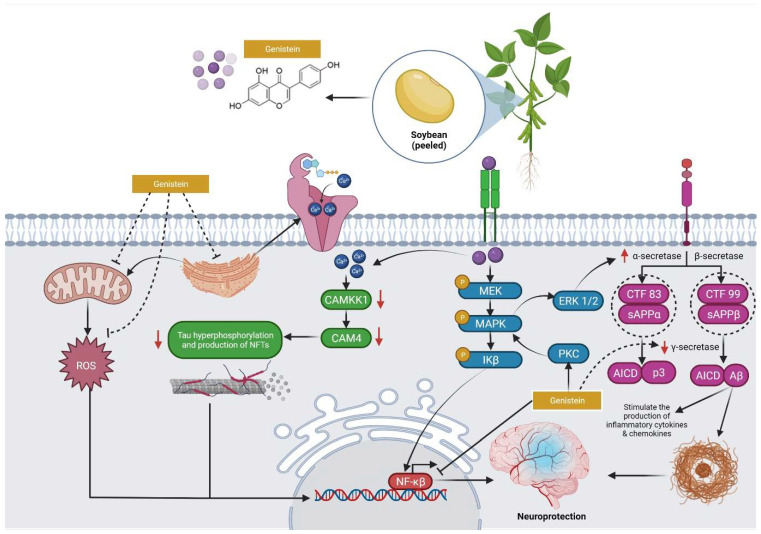
Neuroprotective effects of genistein. The soy isoflavone genistein, which can interact directly with the targeted signalling proteins and maintain their activity to counteract the progression of Alzheimer’s disease, may also help to ameliorate brain deficits caused by Aβ. Abbreviations: CAMKK1, Calcium/calmodulin-dependent protein kinase kinase 1; CAM4, Calmodulin-4; MAPK, Mitogen-activated protein kinase; Ikβ, ERK 1/2, Extracellular signal-regulated kinase 1/2; PKC, Protein kinase C; CTF 83 & 99, CCAAT box-binding transcription factor 83 & 99; sAPPα, Soluble amyloid protein procurer alpha; sAPPβ, Soluble amyloid protein procurer beta; AICD, Amyloid precursor protein Intracellular cytoplasmic/C-terminal domain; Aβ, Amyloid beta; ROS, Reactive oxygen species; NFTs, Neurofibrillary tangles; NF-κβ, Nuclear factor kappa light chain enhancer of activated B cells.

The pharmacological properties of genistein revealed that it has the potential to be a lead molecule in the treatment of a wide range of diseases, including postmenopausal symptoms, cancer, bone, brain, and heart problems [10]. Since genistein is believed to pass the blood–brain barrier to exert its neuroprotective effect, it is extensively applied in the investigation of the treatment of neurodegenerative diseases, such as Alzheimer’s, Huntington’s, and Sanfilippo disease (Figure 3) [11,12,13]. Recent investigations have focused on its effect on MI where genistein protects against MI by (1) reducing the production of β-amyloid protein (Aβ), (2) preventing neuro-inflammatory by inhibiting nuclear factor-activated B cells (NF-κB), (3) inhibiting the activity of acetylcholinesterase (AChE), (4) decreasing the hyperphosphorylation of tau protein to prevent neuronal fiber entanglement (NFT), (5) up-regulating the activity of Apolipoprotein E (ApoE) to reduce the deposition of Aβ, and (6) exerting its antioxidant properties and reducing oxidative stress by eliminating reactive oxygen species (ROS) [14,15,16,17,18,19].

An overview of numerous studies on genistein against MI is offered in this review to better understand the potential functions of genistein in ameliorating MI. The study design of each important work on MI, in terms of animal models, memory testing methodologies, and its dose, is also summarized. Additionally, an overview of the data gathered on the effectiveness of genistein in the treatment of MI is presented. The potential protective mechanisms imparted by genistein are also highlighted to close the knowledge gap, regarding its use as a complementary medicine or an adjuvant for MI. This review also outlines some of the obstacles to, and the potential for improving genistein drug delivery for MI treatment. In addition, various structural modifications and derivatives of genistein were discussed in order to increase its safety, efficacy, physicochemical, and drug-likeness properties.

## 2. Description of Study Design

### 2.1. Animals

Rats and mice were used in the majority of investigations. All experimental protocols were authorized by the relevant institution’s animal welfare committees and conducted in accordance with the guidelines for the use and care of laboratory animals.

### 2.2. MI Models

Each included study focused on different types of MI models. For example, Rumman et al. [19] focused on hypoxia-induced MI while Lu et al. [20] scrutinized on chronic sleep deprivation (CSD)-induced memory deficits. Two studies employed a streptozotocin (STZ)-induced model of MI, where Pierzynowska et al. [13] investigated a STZ-induced Alzheimer’s disease (AD) model while Rajput et al. [21] focused on STZ-induced diabetes for an MI model. On the other hand, other MI models included scopolamine-induced cognitive impairment, lipopolysaccharide (LPS), lead, kainic acid (KA), aging, and β-amyloid [22,23,24,25,26,27].

### 2.3. Genistein Dose

During the course of the treatment, all of the selected investigations used purchased genistein (purity > 98%). The majority of the investigations utilised genistein at 0.5–150 mg/kg. The two most common doses chosen in MI in-vivo models were 10 and 20 mg/kg. In terms of route of administration, eight investigations utilised oral (p.o.) genistein, while in two studies it was given intraperitoneally (i.p.). Prior to the behavioral assessments, genistein treatment was performed for a minimum of 4 days and a maximum of 90 days.

### 2.4. Toxicity Profile of Genistein

An in vivo study, Ek et al. [28] investigated the toxicity profile and pharmacokinetics of genistein in mice. Female BALB/c mice were used during the studies, in which each mouse received intraperitoneal injection with 0.2 mL (10%) dimethylsulphoxide/phosphate buffer solution (DMSO/PBS) containing either 0, 2, 20, 200, 400, and 800 µg of genistein daily for 10 days. Subsequently, the mice were monitored for 14 days, following which any surviving mice were sacrificed for histology analysis. The findings indicated that the mice treated with genistein did not show any signs of toxicity, nor did they become frail, lethargic, or lose any weight, even following treatment with the highest dose of genistein (40 mg/kg). Further, genistein was well tolerated in the in vivo subchronic and chronic safety investigations at doses up to 500 mg/kg/day administered orally for up to 52 weeks according to Nasri and Pohjanvirta [29].

### 2.5. Memory Testing Procedure

Levin and Buccafusco [30] stated that there are three main major cognitive dysfunctions in animal model studies, namely (1) pharmacological models, (2) toxicological models, and (3) genetically modified models. Neural bases of learning, memory, and attention were determined by using critically significant animal models of cognitive impairment. Pharmacological models are the most widely used in cognitive disorders studies since they provide the basis for understanding the role of the neurotransmitter-receptor system involved in cognitive processes, such as learning, memory, and attention [30].

The cholinergic system (muscarinic and nicotinic) and glutamate receptors, mostly the N-methyl-D-aspartate (NMDA) receptors play critical neuronal role in the cognitive functions. Acetylcholine is synthesized from the dietary choline and acetyl coenzyme A via the enzyme choline acetyltransferase (CAT). The metabolism of acetylcholine occurs in the neuronal synapse, facilitated by the enzyme acetylcholinesterase. To date, some cholinesterase inhibitors have been developed to ameliorate impaired memory, such as donepezil, rivastigmine, and galantamine [31].

To induce memory impairment in cholinergic system animal models, antimuscarinic agents, including scopolamine, atropine, pirenzepine, trihexyphenidyl, benztropine, biperidine, and dicyclomine [32], have been utilised. Nicotinic receptor antagonists, such as mecamylamine (a non-competitive nonselective nicotinic receptor antagonist), chlorisondamine and d-tubocurarine (non-specific nicotinic antagonists), dihydro-β-erythroidine hydrobromide (DhβE; a specific receptor α4β2 antagonist), and methyllycaconitine (MLA) (specific receptor α7 antagonist), have all been utilised to stimulate cognitive defects in animal models [33]. Similarly, NMDA receptors also play a critical role in cognitive functions, since their activation is associated with a long-term potentiation (LTP) to strengthen the signal transmission between neurons. Therefore, to stimulate cognitive impairment in animal models through the glutamate-receptor system, many researchers have opted for the use of NMDA receptor antagonists, such as MK-801, ketamine, and phencyclidine (PCP) [34].

Neurological toxicology has been successfully applied in the investigation of cognitive dysfunction in animal models. Neurotoxicity in animal models is achieved by using neurotoxicants, such as lead, mercury, and polychlorinated biphenyls (PCBs), since the cognitive defects have been well-modelled in monkeys and rodents models [30]. Lead, in particular, has been reported in many studies to induce oxidative stress. It induces oxidative stress by increasing vulnerability towards reactive oxygen species (ROS) and reducing antioxidants such as catalase (CAT) and superoxide dismutase (SOD). ROS are mostly generated by protein kinase C (PKC) and nuclear factor-activated B cells (NF-κB), which can be induced by lead exposure. Lead can also cause neuronal cell apoptosis by mimicking calcium ions and binding to the voltage-gated calcium ion channels, thus affecting the neurotransmitter balance in the hippocampus which can cause apoptosis and autophagy. Finally, lead can also induce neuro-inflammatory reaction by activating NF-κB [24].

**Figure 4:**
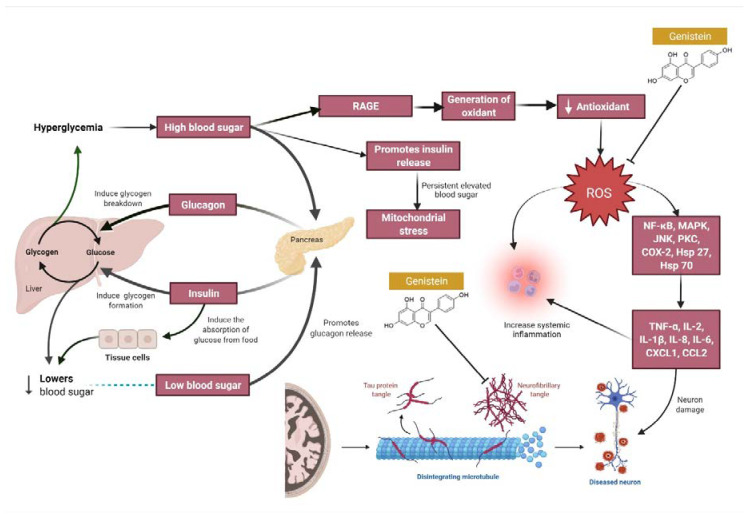
Hyperglycemia and its consequences to neurons. Hyperglycemia produces systemic inflammation and continuous cycles of oxidative and mitochondrial stress, as well as activates multiple downstream kinases that drive a cascade of cytokine and chemokine release, producing additional neuronal damage. Genistein aids in the inhibition of reactive oxygen species (ROS) and the aggregation of Tau protein tangles, minimising the risk of neuronal injury. Abbreviations: NF-κβ, Nuclear factor kappa light chain enhancer of activated B cells; MAPK, Mitogen-activated protein kinase; JNK, c-Jun N-terminal kinase, PKC, Protein kinase C; ROS, Reactive oxygen species; COX-2, Cyclooxygenase-2; Hsp 27 & 70, Heat shock protein 27 & 70; TNF-α, Tumour necrosis factor alpha; IL-2, 1β, 8 & 6, Interleukin-2, 1 beta, 8 & 6; CXCL1, C-X-C motif chemokine ligand 1; CCL, C-C motif chemokine ligand 2.

Streptozotocin (STZ) is widely used in the induction of diabetes in animal models of Alzheimer’s disease (AD). Intracerebroventricular injection of STZ induces hyperphosphorylation of tau protein and accumulation of β-amyloid which can lead to MI [13]. STZ is also used to induce a diabetic state in animal models to stimulate MI by causing hyperglycemia and hypoinsulinemia. Consistently high blood glucose induces inflammation and oxidative stress, as well as activating multiple downstream kinases that activate the release pro-inflammatory cytokines, such as IL-6, IL-1β, and TNF-α, further damaging neurons (Figure 4). Although STZ can cause significant weight loss in animal models, aligned with a major symptom of hyperglycemia, treatment with antihyperglycemics and insulin sensitizers can ameliorate the cognitive deficits [35].

Genetically modified animal models are increasingly used in cognitive impairment studies since they can mimic certain defects, including Alzheimer’s disease (AD), β-amyloid deposition, amyloid precursor protein (APP), and cholinergic-receptor knockout, to be employed for new drug development [30]. On the other hand, memory is accessed using different experimental procedures, including (1) Morris water maze (MWM), (2) passive avoidance test (PAT), (3) novel object recognition (NOR), (4) object location recognition (OLR), (5) novel object discrimination (NOD), (6) elevated plus maze (EPM), (7) delayed spatial alternation (DSA), (8) differential reinforcement of low rates of responding (DRL), (9) Radial arm maze (RAM) task, and (10) Y-maze. Among these studies, MWM, NOR, and OLR are the three most commonly used method for memory testing.

#### 2.5.1. Morris Water Maze (MWM)

The Morris water maze (MWM) is a circular, water-containing steel pool with differing diameter and height, ranging from 100–160 cm in diameter and 38–80 cm in height. The pool is divided into four similar quadrants (marked as NE, SE, NW, and SW) and a platform will be submerged underwater in the middle of one of the stated quadrants [19,22]. The platform is kept at the same place throughout the testing session.

The animals are trained for a few days to determine the location of the platform. During training, they are released from different quadrants while facing the quadrants’ wall and will swim to towards the submerged platform for 60, 90, or 120 s. If the animals fail to find the platform during the allocated time, they will be placed onto the platform for another 10 or 30 s to make them feel familiar. The training phase will continue for a few days before the actual trial where a harmless opaque ink will be put inside the pool to hide the location of the platform [22]. The animals will be given a certain time to determine the location of the submerged hidden platform. The time taken will be recorded to assess the long term spatial memory. Another assessment can also be performed to evaluate memory retention, in which during the trial phase, the platform will be removed and the number of animal crossing on the target platform former location will be recorded using a video camera [24].

#### 2.5.2. Passive Avoidance Task (PAT)

The passive avoidance task (PAT) involves the use of an apparatus that is divided into illuminated and dark compartments that are connected by a small gate. During the acclimatization phase, the animals are placed inside the apparatus for 15 min to familiarize themselves of the new environment. During the training trial, the animals will be put inside a dark compartment, and a small electric shock (39 V for 3 s or 1 mA for 1 s) will be released to their feet. After 24 h of the training trial, the actual test will be performed during which each animal will be placed in the illuminated compartment. The latency period before it entered the dark compartment will be recorded up to a maximum of 300 s to assess the animals’ retention memory of the electric shock in avoiding the dark compartment when they received the shock [19,27].

#### 2.5.3. Novel Object Recognition (NOR)

The novel object recognition test (NOR) is performed to assess the animals’ recognition memory. The test is performed in a rectangular box (40 cm × 50 cm × 50 cm) that is painted in black with a video camera set up above the chamber to record the animals’ behaviour. In the habituation phase, the animals are placed inside the chamber without the presence of any objects for at least 10 min for three consecutive days. During the trial phase, the animals will be allowed to roam inside the box containing two identical objects (typically plastic balls) for 5 min. After 30 min, the test trial will be performed where one of the objects will be replaced with another object of different color. The exploratory behaviour of the animals will be observed based on the sniffing or touching action of the object. The duration of contact with each of the object will be recorded to evaluate the recognition memory [19,20].

#### 2.5.4. Object Location Recognition (OLR)

The object location recognition test is used to assess the recognition memory, which is similar with the NOR test. The apparatus is a rectangular box (40 × 50 × 50 cm) with a dark painted chamber inside and a video camera mounted on top of the chamber to observe the animals’ exploratory behaviour. The objects used are two small plastic bottles, identical in size and shape but different in color. The method is divided into three phases: habituation, familiarization and test phases [20].

Habituation phase: The animals are allowed to roam freely inside the chamber with no objects for 10 min for three consecutive days.

Familiarization phase: On the fourth day, the animals are placed inside the chamber containing two identical objects for 5 min.

Test phase: 30 min after the familiarization phase ended, the animals will be placed inside the chamber again, but one of the original objects will be replaced with a different object while the remaining original object is still kept inside the chamber.

To avoid any possible odor cues, the objects and the floor of the chamber are cleaned using 70% ethanol at the end of each trial session. The animals’ exploratory behaviour during the test phase are observed based on the sniffing or touching action of the object using the animals’ nose [22].

#### 2.5.5. Novel Object Discrimination (NOD)

The novel object discrimination test allows the animals to explore two objects for 5 min during the familiarization phase. After 4 h, one of the objects will be replaced with a new one. Subsequently, the exploratory behaviour of the animals, such as chewing, licking, sniffing, or touching the object with their noses [23], will be recorded.

#### 2.5.6. Elevated Plus Maze (EPM)

The elevated plus maze involves the use of an elevated plus-shaped apparatus which consists of four elongated rails (arms), two of which are open arms and the other two, enclosed arms. The two open arms are situated across each other, perpendicular to the enclosed arms with a platform in the middle [36]. During the training phase, the animals are placed at the end of the open arm, facing away from central platform for three days. The transfer latency time (TLT) is recorded as the time taken by the animals to enter the enclosed arm from the starting point on the open arm within 90 s. On the fourth day, during the test trial, the TLT is recorded 24 h after the global cerebral ischemia-reperfusion (IR) brain damage, which is the index for memory [21].

#### 2.5.7. Delayed Spatial Alternation (DSA)/Differential Reinforcement of Low Rates of Responding (DRL)

The delayed spatial alternation (DSA) and differential reinforcement of low rates of responding (DRL) involves the use of similar apparatus, a Skinner box, containing two retractable levers between a pellet dispensers with a pair of cue lights directly above each levers. During the training phase, the animals are trained to press the lever based on the cue light for food pellets as a reinforcer, based on an autoshaping program. To prevent the animals from developing a side bias towards the lever, the lever associated with the reinforcement is interspersed with every five reinforcers delivered. The DSA task involved a delay in the lever press for 0, 3, 6, 9, or 18 s. The slow responding criteria are divided into six main training sessions, the first two sessions entailing a fixed-ratio 1 schedule for 200 trials or 90 min. The third and fourth sessions composed of DRL-5 s schedule, while reinforcement is dependent on a 5 s delay between responses. The same is applied to the last two main sessions with DRL-10 s schedule that requires a 10 s delay between responses. The animals are tested on DRL-15 s schedule for at least 30 sessions [26].

#### 2.5.8. RAM Task

The radial arm maze (RAM) task is employed to assess the spatial memory and involves the use of an elevated eight-armed radial maze with each arms extended from the octagonal central platform. At the end of each arm, a food container is available for the experimenter to deposit food for reinforcement. Nevertheless, during the trial phase, only some of the arms will contain food pellet in the food container.

During the training phase, the animals will be placed at the central platform and will be allowed to freely explore the maze to acquire food pellet. During the process, the animals will learn not to re-enter the arms that they have visited in the same trial in the absence of food. In the test trial, 10 min will be given for the animals to explore the maze and to consume all the pellets placed in some of the arms. The correct and incorrect choices are used to evaluate each of the animals’ performances. Should the animals re-enter the arms without food that they have visited, it will be considered as an error [27].

#### 2.5.9. Y-Maze

The Y-maze test is used by Bagheri et al. [27] and Shahmohammadi et al. [23] to assess the spatial recognition memory of the animals. The apparatus used is a three-armed maze, where each arm is 120° from another and resembles the shape of a capital “Y”. Each of the arms are connected by an interconnecting part. The protocol was conducted to evaluate spatial learning using the spontaneous alternation in which the animals are naïve to the nature of the maze. The animals are placed at the end of one arm and will be allowed to move freely in an 8 min session. Alternations are observed as successful entries into each of the three arms on overlapping triplet sets, each one with non-repetitive arms. The alternations percentage is subsequently calculated as the ratio of actual to possible alternations × 100.

## 3. Effectiveness of Genistein

### 3.1. Hypoxia

In a study by Rumman et al. [19] impact of genistein on hypoxia-induced MI was investigated using male Swiss albino mice. The mice are continuously treated with 10, 20 or 30 mg/kg/day of genistein p.o. for 28 days. A mice model for amnesia was developed based on hypoxia, by exposing the mice to low level of oxygen (10%) daily for similar duration as that for genistein treatment. Morris water maze (MWM), passive avoidance test (PAT), and novel object recognition (NOR) were used to investigate the effects of genistein in ameliorating memory defects in amnesiac mice.

The results based on MWM showed that mice treated with genistein dose of 20 and 30 mg/kg exhibited a low latency and increase in crossing number at the platform quadrant. As for PAT, there was an increase latency in the 20 and 30 mg/kg genistein groups. Lastly, in NOR, both groups of mice which received 20 and 30 mg/kg genistein showed an increase in exploratory behaviour of the novel object as compared with that for the familiar object. Overall, the findings suggested that treatment with genistein can help reduce memory defects in hypoxia-induced MI.

### 3.2. Chronic Sleep Deprivation (CSD)

In another study by Lu et al. [20] the effects of genistein on chronic sleep deprivation (CSD)-induced MI was investigated in male Institute of Cancer Research (ICR) mice. The mice were treated with genistein (10, 20, or 40 mg/kg/day) p.o. daily for 23 days. Induction of CSD was performed using an automated sleep interruption apparatus (SIA) which consisted of a stainless steel rotator that rotates for 1 min after a 2 min pause for 24 h/day for a total of 14 days. Morris water maze (MWM), object location recognition (OLR), and novel object recognition (NOR) were used to assess the spatial and recognition memory of the CSD-induced mice.

For MWM, the genistein-treated group especially 40 mg/kg group had a significantly decreased the latency in finding the submerged platform. Additionally, in the probe test where the platform was removed, there was a considerable increase in the crossing numbers in the target quadrant among the genistein 20 and 40 mg/kg. The genistein-treated group (20 and 40 mg/kg) showed a notably increased discrimination index (DI) as compared with CSD group in OLR. In the NOR task, there was a significant elevation in the DI, especially among the genistein 20 and 40 mg/kg treatment groups. Overall, genistein treatment (especially 20 and 40 mg/kg) is effective in alleviating memory defects induced by CSD.

### 3.3. Streptozotocin (STZ)

To investigate the neuroprotective effect of genistein against streptozotocin (STZ)-induced cognitive dysfunction, male Wistar rats were administered with streptozotocin (STZ) via an intracerebroventricular (i.c.v.) injection for a cumulative 3 mg/kg over two injections with a 48 h interval [13]. The rats were treated with genistein 150 mg/kg/day p.o. for 90 days. Genistein-treated group showed lower latency to swim towards the platform during the Morris water maze (MWM) test trial. However, in the probe test, the time spent by the genistein-treated rats in the target quadrant was significantly longer than the other groups, indicating that genistein treatment showed promising results in ameliorating STZ-induced MI.

### 3.4. Scopolamine

Lu et al. [22] investigated the effects genistein on scopolamine-induced MI in male Institute of Cancer Research mice. The mice were intraperitoneally (i.p.) administered with scopolamine 0.75 mg/kg/day for seven consecutive days. Genistein (10, 20, or 40 mg/kg/day, p.o.) was administered daily to the mice for 24 days. The behavioural tests involved were object location recognition (OLR) and Morris water maze (MWM) for evaluation of spatial memory. In the OLR task, genistein-treated group (40 mg/kg) showed a significant increase in the discrimination index (DI). In both the trial and probe tests of MWM, the genistein-treated group showed a lower escape latency to locate the submerged platform and showed higher crossing numbers in the target quadrant, indicating that genistein treatment can improve cognitive performance.

### 3.5. Lipopolysaccharides (LPS)

Shahmohammadi et al. [23] conducted a study on the effect of genistein treatment on lipopolysaccharide (LPS)-induced neuro-inflammation on male albino Wistar rats. Neuro-inflammation was induced by introducing 500 µg/kg/day LPS (i.p). The subsequent genistein treatment was performed for seven days at 10, 50, or 100 mg/kg/day. Spatial and recognition memories were assessed using a Y-maze, novel object discrimination (NOD) and passive avoidance task (PAT). In all three tests, genistein (50 and 100 mg/kg) yielded significant improvements in the parameters involved which further support that genistein treatment can alleviate cognitive dysfunction.

### 3.6. Streptozotocin (STZ)-Induced Diabetes

An in vivo study was conducted by Rajput et al. [21] to investigate the neuroprotective role of genistein on STZ-induced diabetes on male albino Swiss mice. Diabetes in the mice was induced by introducing 200 mg/kg of STZ through an i.p. route. Diabetes was induced to cause hyperglycaemia in the mice, which in turn can cause ischemia-reperfusion (IR)-induced neuronal damage. Subsequently, genistein treatment was administered through i.p. to the diabetic mice (2.5, 5, or 10 mg/kg/day) for 14 days. Spatial and retention memories were then evaluated by using an elevated plus maze (EPM) which resulted in a decline in the transfer latency time for the genistein (5 and 10 mg/kg) treatment groups in diabetic mice with IR. Overall, the findings strongly suggest that cognitive deficits can be reduced with genistein treatment.

### 3.7. Lead

Su et al. [24] evaluated the protective effect of genistein treatment on lead level as a toxicant. Male Sprague-Dawley rats were orally administered (p.o.) with both lead and genistein at 1 mg/kg/day for 56 days. A Morris water maze (MWM) was used to evaluate the cognitive performance of the rats and lead influence. Genistein treatment significantly decreased the latency to the platform and caused higher crossing numbers in the target quadrant in both trial and probe tests, thus suggesting that genistein treatment can reduce the effect of MI.

### 3.8. Kainic Acid (KA)-Induced Seizure

In a study by Khodamoradi et al. [25], genistein treatment was investigated for its possible effect on kainic acid (KA)-induced seizure on female Wistar rats. KA-induced seizure resulted in MI and neuronal injuries. KA was administered to the rats via intracerebroventricular (i.c.v.) route (0.5 μg/μL). Subsequently, the introduction of KA to the rats was performed four days after genistein treatment at 0.5 and 5 mg/kg/day through i.p. A Morris water maze (MWM) was used to assess the spatial memory. Overall, the results suggest positive effects of the genistein towards the KA-induced seizure mice.

### 3.9. Aging

Neese et al. [26] investigated the aging-related cognitive deficits and studied the potential protective effect of genistein in alleviating the deficits. They utilized 14-month-old female Long-Evans rats to simulate the aging effects on cognitive performance. Both lever press and Skinner box delayed spatial alternation (DSA) and differential reinforcement of low rates of responding (DRL) were used to evaluate the working memory. Nevertheless, the results indicated that genistein is not effective in ameliorating cognitive deficits in an aged rat MI model.

### 3.10. β-. Amyloid

Bagheri et al. [27] scrutinized the neuroprotective effect of genistein treatment on β-amyloid induced MI. β-amyloid 1–40 was injected i.c.v. (4 μL) into male Wistar rats, followed by the oral introduction of genistein (10 mg/kg/day). Y-maze, passive avoidance test (PAT), and radial arm maze (RAM) tests were used to evaluate the cognitive performance in which the rats treated with genistein showed significant parameter increments in both Y-maze and PAT, while in RAM, there was no significant increase in the correct choices of arms or decrease in incorrect arm choices. Overall, the findings suggested that treatment with genistein can prevent β-amyloid induced MI.

## 4. Overview of the Mechanisms of Action of Genistein against MI

A number of in vivo evidence-based studies have reported that MI can be caused by a plethora of factors including aging [26], unhealthy lifestyles (i.e., chronic sleep deprivation) [20], neurotoxicants (i.e., lead and STZ) [13,24], neurodegenerative disorders (i.e., AD caused by LPS and β-amyloid) [23,27] and certain non-contagious disease (i.e., diabetes) [21]. The effectiveness of genistein in improving cognitive performances, especially in memory domains, including spatial, recognition, retention, and reference memories, have been reported in several of these studies (Table 1). Overall, genistein confers protection against MI by reducing oxidative stress [19,20,21,22,23,24,25,27], attenuating neuro-inflammation [19,20,23], enhancing cholinergic neurotransmission [22,27], degrading pathological proteins [13], preventing apoptosis [21], and increasing the expression neuroprotective genes (CREB, CBP, BDNF, IGF-1, and ERK) [19,22].

The overproduction of reactive species especially reactive oxygen species (ROS) and reactive nitrogen species (RNS) coupled with the failure of antioxidant system in the body can cause a cascade of cellular destruction, including neuronal damage [37]. When ROS and RNS levels exceed the scavenging capacity of antioxidants in the body, oxidative stress occurs, which is harmful to the cellular functions, and especially cognitive performance. The relationship between the onset of neurodegenerative disorders (NDD) (i.e., AD, PD, and HD) is closely linked with oxidative stress and its effect on the formation of neuronal plaques, neurofibrillary tangles (NFTs) as well as the formation of β-amyloid following the overproduction of ROS closely associated with the NDD [38]. In fact, the brain is said to be the most vulnerable to oxidative stress because of its particularly high oxygen consumption, high ROS production by the mitochondria, and low antioxidant capacity [20]. Genistein, an isoflavone mainly found in soybean, has confirmed to have the ability to alleviate the deleterious effects of oxidative stress on neuronal injury, such as preventing neuronal death [21], increasing the production of hippocampal glutathione (GSH) and superoxide dismutase (SOD) [20], and lowering lipid peroxidation, ROS, and nitric oxide production [19].

The activation of the neuro-immune cells (microglia and astrocytes) into pro-inflammatory states (or also known as neuro-inflammation) is closely linked with the pathophysiology of NDD including AD [39]. Shahmohammadi et al. [23] and Lu et al. [20] respectively reported that both lipopolysaccharides (LPS) injection and sleep deprivation (SD) can lead to an increased level of pro-inflammatory cytokines and mediators, such as tumor necrosis factor α (TNF-α), nuclear factor activated B-cell (NF-κB), toll-like receptor 4 (TLR4), and interleukin 6 (IL-6). The over-expression of TLR4 will activate neuro-inflammation signalling and increase the TLR4 level, which can worsen the memory processes in the hippocampus. Activation of NF-κB also mediates the production of other pro-inflammatory cytokines, including inducible nitric oxide synthase (iNOS) and cyclooxygenase-2 (COX-2), which can induce neuro-inflammatory reactions [40]. In β-amyloid MI models, there is a decline in the activity of choline acetyltransferase (ChAT) in the cerebral cortex and hippocampus, along with the decreased production of acetylcholine (Ach) and dopamine [27]. The dopaminergic–cholinergic system is closely linked with the mechanism of NDD and a decline in dopaminergic action which will lead to striatal cholinergic interneuron (SCI) over-activation. This phenomenon, in turn contributes to a reduction in the release of dopamine. Additionally, both the degeneration of cholinergic and dopaminergic neurons are closely involved in the development of NDD [41]. In scopolamine MI models, genistein treatment significantly reduced acetylcholinesterase (AChE), increased ChAT activities, and increased the expression of Ach in the hippocampus compared with scopolamine models untreated with genistein, thus indicating the potential of genistein in increasing AChE activity and lowering Ach levels [22].

In many NDD, the pathological process includes the formation of pathological protein, such as β-amyloid 1–42 and tau protein, while recent studies suggest utilisation of autophagy to degrade these pathological protein in order to effectively treat NDD [12]. In another study, a high dose of genistein (150 mg/kg/day) was administered in STZ-induced AD animal model to stimulate autophagy of pathological proteins associated with AD. Genistein treatment significantly alleviated the memory and behavioural disturbances in the AD animal model, along with reducing the levels of pathological proteins in the hippocampus and cerebral cortex. Degradation of pathological proteins through autophagy by the action of genistein is confirmed as shown by the elimination of the primary cause of the disease, which is the accumulation and aggregation of pathological protein [13].

Diabetes-induced cerebral ischemia reperfusion (IR) can trigger apoptosis, leading to neuronal death. Rajput et al. [21] investigated the effect of genistein in preventing neural cells apoptosis in STZ-induced diabetic animal MI model. Genistein helps in obstructing neuronal apoptosis through some mechanisms, including (1) reducing the production and aggregation of β-amyloid, (2) reducing the phosphorylation of tau protein, and (3) eliminating ROS and free radicals, all of which are closely linked with neuronal apoptosis [42]. The transcription of factors, such as brain derived neurotrophic factor (BDNF), cAMP-response element binding protein (CREB), CREB-binding protein (CBP), insulin-like growth factor-1 (IGF-1), and extracellular signal-regulated kinase (ERK), are closely related with cognitive performance, especially with memory.

BDNF is a growth factor closely linked with the development and proliferation of neural cells and disturbance in BDNF levels would result in memory dysfunction. Memory disorder is also correlated with the perturbation of CREB levels. The main function of IGF-1 is to ameliorate the effect of growth hormone (GH) in humans. Its other effect concerns the proliferation of neurons in the brain, where the under-expression of IGF-1 is closely linked with memory impairment. Additionally, ERK plays an important role in synaptic plasticity and the development of memory domains [19,22].

The reported studies used a minimum of 0.5 mg/kg and a maximum of 150 mg/kg dose of genistein with treatment duration ranging from 4 to 90 days. Based on our review of the literature, we noticed that the obtained results were not compared to standard drugs. However, on the other hand, preclinical investigations strongly suggested that genistein is effective in enhancing the cognitive function of MI animal models, even at a low dose level, thus indicating its potential efficacy for treating MI.

## 5. Pharmacokinetics and Bioavailability of Genistein

Due to its low molecular weight and lipophilic property, genistein is rapidly absorbed into the body. Yang et al. [43] reported a high absorption (77%) of genistein in rat/mouse while the lowest absorption reported was 28% where genistein showed different absorption depending on the region of the intestinal tract; duodenum (44%), colon (35%), ileum (18%), and jejunum (16%). Interestingly, there are gender differences in its absorption, as female rats had a relatively complete absorption (~100%) of genistein as compared to male rats (56%). Nevertheless, it is unclear why the discrepancies occur.

Jaiswal et al. [44] stated that orally, only 20–40% of genistein is absorbed which eventually contributes to the low bioavailability, with time to the maximal absorption (T_max_) at 5–6 h and a half-life of 8 h. Kwon et al. [45] reported that genistin, which is the glycoside form of genistein that is in a free form, has a greater bioavailability following oral administration. The occurrence is most likely due to the ability of genistin to be absorbed in both forms (genisten and genistein) in the intestinal tract where the former can passively be transported across the enterocytes membrane and directly enter the circulation with the help of a sodium dependent glucose transporter (SGLT1). Genistein is rapidly distributed throughout the body and is abundant mostly in the gastrointestinal tract (18.5 μg/g) and liver (0.98 μg/g). The half maximal effective concentration (EC_50_) of genistein exceeded in other tissues suggesting a better competition with estradiol to activate the estrogen receptors (ERα and ERβ).

Genistein, one of the dietary isoflavones, is metabolized in the intestine, which includes reactions such as oxidation, reduction, and conjugation. In fact, the metabolism of genistein is closely associated with the metabolism of endogenous estrogen, in which phase 2 (conjugation) reactions are predominantly higher as compared with phase 1 (oxidation and reduction) metabolic reactions. The two major metabolic pathways for genistein are glucuronidation and sulfation, with the enzymes UDP glucuronosyl transferase (UDT) and sulfotransferase (SULT) mediating the conjugation reactions. Yang et al. [43] reported a high metabolic rate of genistein in the intestine following oral, intravenous, and intraperitoneal administration. Due to the high activity of UDT and SULT in the intestine, most of the dietary genistein are metabolized in the enterocytes before being transported to the liver where the remaining aglycone genistein is metabolized. It was also reported that UDT and SULT are also present in high concentrations in other tissues (kidney, heart, lung) indicating the possibility of genistein being metabolized in these sites. Nevertheless, the main sites for genistein excretion are through biliary and renal excretions. In the urine, genistein is mainly excreted in its conjugated forms, i.e., monoglucuronide (53–76%), diglucuronide (12–16%), and sulfoglucuronide (2–15%). Overall, genistein is mainly excreted in the glucuronide form compared to its aglycone, as evidenced by the high concentration of glucuronide genistein in the bile [44].

## 6. Challenges and Opportunities to Improve the Drug Delivery of Genistein for MI

**Figure 5:**
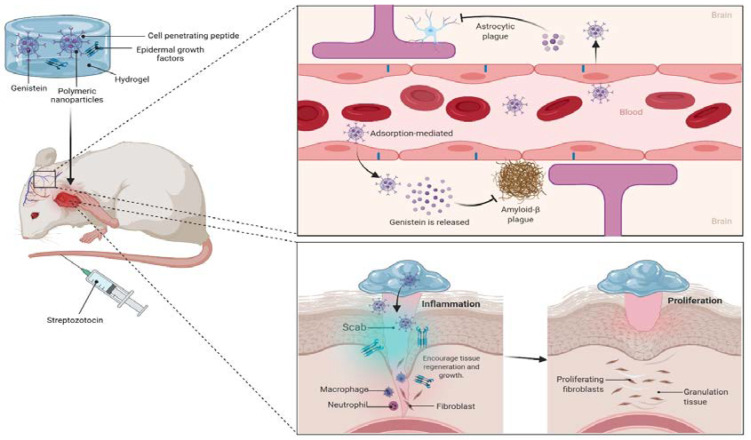
Future perspectives of hydrogel loaded epidermal growth factors with genistein-polymeric nanoparticles for effective wound healing and inhibition of amyloid beta plague.

The data acquired from DruLiTo software [46] indicate that genistein is a good potential drug-like molecule and can be a good therapeutic agent for a variety of disorders including neurodegenerative disorders (Table 2). Nevertheless, despite its promising therapeutic activity against MI as confirmed in several in vivo animal models studies, confirming its potential against MI in human clinical trials remains a challenging obstacle. Several difficulties hindering its clinical effectiveness include its poor water solubility, insufficient targeting of pathological protein, rapid metabolism and excretion, as well as low bioavailability following oral administration. Additionally, genistein can cross the blood–brain barrier (BBB) rapidly easily, making the brain another of its site of action (Figure 5) [44]. Although genistein is widely found in leguminous plants mainly in soybean, which is commonly consumed, phase I clinical trials can be commenced without any problems as soybean is deemed to be safe for human consumption and has been used in many traditional cuisines, especially in the Asian regions [8].

Low serum level of genistein following oral administration is a disadvantage, restricting its transport to the site of action and for exertion of pharmacological actions. The use of nanoformulation to deliver genistein into the body may be an effective method to enhance its water solubility, permeability, bioavailability, as well as its overall therapeutic response. In addition to nanoformulation, other genistein formulation systems can also be considered to enhance its delivery into the human body, including administering as tablets (facilitates the immediate release of genistein), solid lipid microparticles (to enhance bioavailability), microparticles (to enhance water solubility and bioavailability), solid lipid nanoparticles (to enhance bioavailability), hydrogel matrix (to improve water solubility), micelles (to improve bioavailability after oral administration), nanostructured lipid carriers (to enhance oral bioavailability), and liposomes (to enhance permeability and retention) [44].

In addition, drug metabolism and pharmacokinetics (DMPK) research is crucial in order to comprehend both the efficacy and safety of genistein against NDD and its indirect protective effect against MI. Genistein is structurally modifiable to enhance its DMPK properties, along with amplifying its overall therapeutic properties including improving its aqueous solubility, increasing permeability and retention as well as ameliorating toxicity and adverse reactions. Finally, the mutual cooperation and understanding between experts in medicinal and organic chemistries are essential in modifying genistein for therapeutic and commercial use through drug research and development. The findings can be used to develop a potential natural lead compound for drug design and development in treating MI.

## 7. Possible Structural Modifications and Derivatives of Genistein

Chemists have been prompted to develop many derivatives of genistein (**1**) because of the recognised biological benefits. A series of naturally occurring genistein (**1**) and biochanin A (**2**) molecules, as well as their analogues (**4**–**6**) were synthesised and evaluated for the antioxidant activity [47]. The 7-O-carboxymethyl-genistein (**8**) was synthesised from genistein (**1)** or from sophoricoside (**7**) where the carboxylate group increased the water solubility of genistein (**1**) by more than a thousand-fold that are reported as valuable drug-like candidates in pharmacological actions [48].

The cytotoxic actions of the synthesised genistein amino acid derivatives (**9a**–**9d**) were in fact reported to be more effective than genistein (**1**) [49]. The 6-carboxymethyl genistein (**10**) has been identified as a new synthetic oestrogen receptor modulator and an excellent substrate for building conjugates with anthracyclines [50,51]. Additionally, the antiosteoporotic and anti-proliferative properties of the derivatives (**11a**–**e**) have been described by a group of researchers [52,53]. Further, to improve solubility, a genistein-piperazine complex (**12**) was developed, in which the hydroxyl group at C-7 of genistein (**1**) was hydrogen-bonded to the nitrogen of piperazine [54].

To boost genistein’s biological activity by enhancing its cellular absorption rate and extending its stability as well as blood circulation, Meng et al. [55] identified a number of genistein fatty acid esters (**13a**–**13c**). On the other hand, Matsumoto et al. [56] developed genistein derivatives (**14a**–**14c**) as nitric oxide donors, which have the potential to inhibit tyrosine kinase activity while also increasing nitric oxide levels. Kohen et al. [50] reported genistein derivatives (**15a** and **15b**) with much more enhanced antiproliferative activity than genistein (**1**). Additionally, 7-O-modified derivatives of genistein (**16a**–**16c**, **17a**, and **17b**) were reported by Zhang et al. [57]

In another study, Li et al. [58] synthesised and investigated deoxybenzoin genistein derivatives for its antibacterial activity, and reported that the dimeric deoxybenzoin (**18a**–**18e**) derivatives are generally more active than genistein (**1**). Besides, Rusin et al. [59] and Rusin et al. [60] discovered a new genistein glycoside (**19**). The glycoside form of genistein (**20a**–**20d**) and O-tetradecanoyl-genistein (**21**) can reduce the clinical manifestations of experimental autoimmune encephalomyelitis, a murine autoimmune disease used to study multiple sclerosis [61]. Furthermore, anti-Alzheimer’s activity has been studied for compounds **22a**–**22c** and **23a**–**23h**. Similarly, cholinesterase inhibition, metal-chelating activity, and human hepatoma cell viability were investigated for a series of new genistein–polyamine conjugates (**24a**–**24h**) [62].

**Figure 6:**
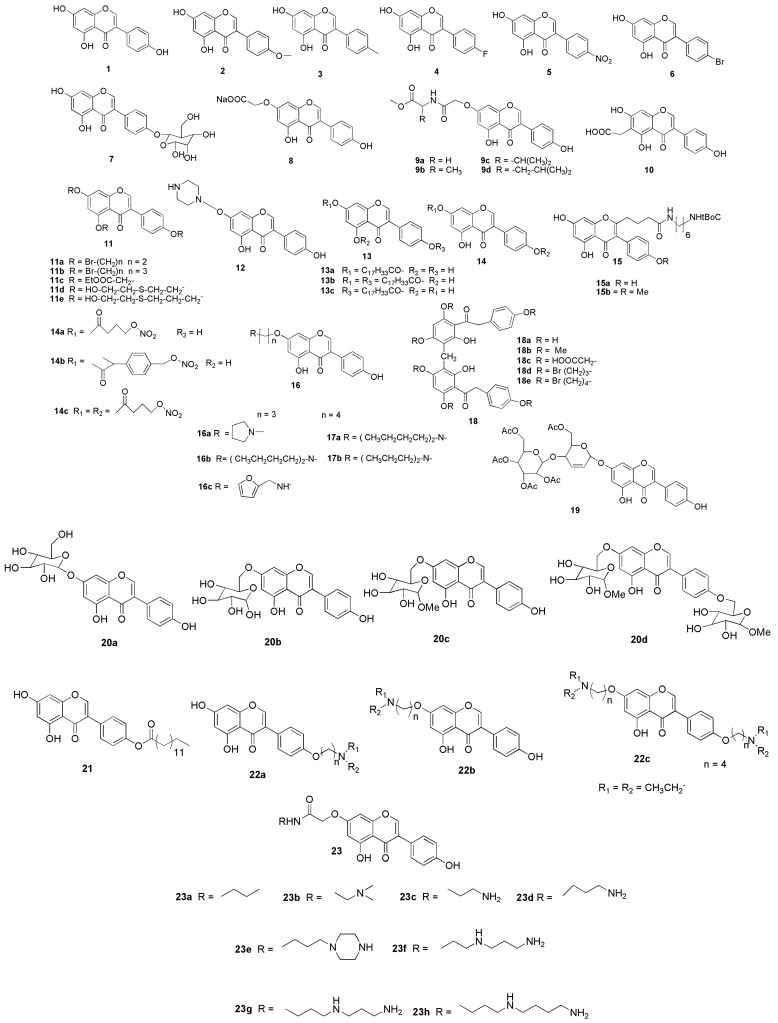
Possible structural modifications and derivatives of genistein.

**Figure 7:**
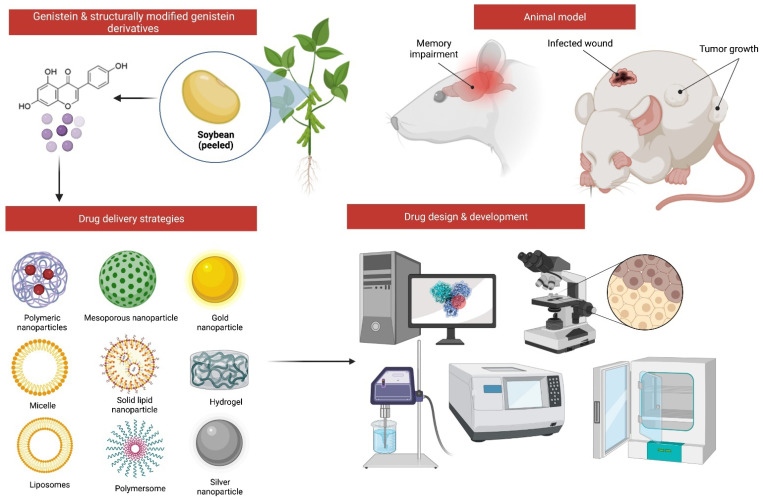
Incorporating structurally modified genistein into a drug delivery system may increase drug delivery to the brain and other targeted areas.

It remains unclear on whether the structural modifications of genistein can improve its physicochemical and biological properties, including its pharmacological actions against MI. Using various chemical processes, many analogues of genistein have also been produced (Figure 6). The synthesis of more potent genistein derived compounds in silico may pave the way for new drug discovery and development. Nonetheless, more in vitro and in vivo research is needed to demonstrate the safety and efficacy of all semisynthetic genistein derivatives. In the future, more research into the structure–activity relationship (SAR) of genistein will be required to obtain various other unique compounds that can be developed from it. In addition to conducting further study in order to better understand the therapeutic abilities of genistein against MI, additional experimentation to support brain targeting drug-delivery of genistein is required (Figure 7). Additionally, the implementation of randomized control trials can further strengthen the claims in this review.

## 8. Conclusions

Overall, genistein is a promising natural lead for improving cognitive performance in all three types of MI models in animals (pharmacological, toxicological, and genetic models). Genistein attenuates neuronal damage via several mechanisms as described in this review and has demonstrated good potential to be investigated as a novel therapeutic agent in the treatment of MI. Conclusively, through this review, it has been implicated that genistein in MI animal models acts mainly through the antioxidant defence mechanism, conferring protection against inflammation, enhancing neurotransmission, inducing autophagy and the prevention of apoptosis, and increasing the expression of neuroprotective genes. An in silico method to select more potent genistein-derived molecules could pave the way for new drug development and discovery. Nonetheless, more in-vitro and in-vivo research is needed to show that all semisynthetic genistein derivatives are safe and effective. More research into the structure–activity relationship (SAR) of genistein will be required in the future to produce a variety of additional unique molecules from it. Altogether, genistein is a potential natural lead for the design and development of a novel neuroprotective drug, in our perspective and based on scientific evidence.

## Figures and Tables

**Table 1:** A summary of in-vivo studies investigated the efficacy of genistein on memory impairment.

Animal, Sex	MI Model	Genistein Dose (mg/kg/day)	Route of Adminis-tration	Duration of Treatment (Days)	Memory Type	Memory Parameters	Remarks	References
Swiss albino mice, male	Hypoxia	10, 20, 30	p.o.	28	Spatial memory, retention memory, recognition memory	MWM, PAT, NOR	Cognitive dysfunction prevented	Rumman et al. [19]
ICR mice, male	CSD	10, 20, 40	p.o.	23	Spatial memory, recognition memory	MWM, OLR, NOR	Deleterious effects alleviated	Lu et al. [20]
Wistar rat, male	STZ	150	p.o.	90	Spatial memory	MWM	Degradation of pathological proteins increased	Pierzynowska et al. [13]
ICR mice, male	Scopolamine	10, 20, 40	p.o.	24	Spatial memory	OLR, MWM	Cognitive performance improved	Lu et al. [22]
Albino Wistar rat, male	LPS	10, 50, 100	p.o.	7	Spatial memory, recognition memory	Y-maze, NOD, PAT	Cognitive dysfunction alleviated	Shahmohammadi et al. [23]
Swiss albino mice, male	STZ-induced diabetes	2.5, 5.0, 10.0	i.p.	14	Spatial memory, retention memory	EPM	Cognitive deficits reduced	Rajput et al. [21]
Sprague-Dawley rat, male	Lead	1	p.o.	56	Spatial memory	MWM	Memory impairment reduced	Su et al. [24]
Wistar rat, female	KA-induced seizure	0.5, 5.0	i.p.	4	Spatial memory	MWM	Memory impairment reduced	Khodamoradi et al. [25]
Long-Evans rat, female	Aging	10.2	p.o.	-	Working memory	DSA, DRL	Cognitive deficits are not affected	Neese et al. [26]
Wistar rat, male	β-amyloid	10	p.o.	-	Spatial memory, recognition memory, working memory, retention memory, reference memory	Y-maze, PAT, RAM	Memory impairment reduced	Bagheri et al. [27]

Abbreviations: ICR, Institute of Cancer Research; CSD, Chronic sleep deprivation; STZ, Streptozotocin; LPS, Lipopolysaccharide; KA, Kainic acid; p.o., Oral administration Per os; i.p., Intraperitoneal injection; MWM, Morris Water Maze; PAT, Passive avoidance test; NOR, Novel object recognition; OLR, Object location recognition; NOD, Novel object discrimination; EPM, Elevated plus maze; DSA, Delayed spatial alternation; DRL, Differential reinforcement of low rates of responding; RAM, Radial arm maze.

**Table 2:** Physicochemical and drug-likeness properties of genistein.

Property/Rule	Result
Molecular formula	C_15_H_10_O_5_
Molecular weight	270.24
Hydrogen bond donors	3
Hydrogen bond acceptors	5
Rotatable bonds	1
Log P (Partition coefficient, predicted value)	1.043
Molar refractivity	78.92 cm^3^
Topological polar surface area	86.99 Å^2^
Lipinski’s rule of five	Passed
Ghose filter	Passed
Veber’s rule	Passed
BBB likeness rule	Passed
Unweighted QED	Passed
Weighted QED	Passed

Abbreviations: BBB, Blood Brain Barrier; QED, Quantitative Estimate of Drug-likeness.

## Data Availability

The data presented in this study are available on request from the corresponding author.
